# Engaging with Records and Archives: Histories and Theories

**DOI:** 10.5195/jmla.2017.268

**Published:** 2017-07-01

**Authors:** Paul M. Blobaum

Archives are typically imagined as a file morgue, that dusty place to which papers, records, and other collections are deposited for permanent storage. The archivist is somewhat of a “person of mystery,” as archivists are not depicted in popular culture and the role of the archivist is not widely under-stood. This book fills in these gaps of knowledge, giving the reader insight into not only the thoughts and discussion of people working in archives, but also the fascinating world of archival collecting and research. This book of scholarly essays from the Seventh International Conference on the History of Records and Archives (I-CHORA 7) held in Amsterdam, Netherlands, in July 2015 covers a wide variety of topics, range of time periods, and international settings. Readers familiar with ongoing theories and controversies in archival practice will find this book informative and a compelling contribution to archival literature.

As the subtitle notes, the book discusses both theory and history of archives in an international context. Part 1, “Rethinking Histories and Theories,” presents theories of archival practice and essays on the history of women archivists in England, archival description, and the concept of “archival silence.” Part 2, “Engaging Records and Archives,” presents histories of the creation and use of archives. The diverse papers submitted by eleven authors make challenging reading, so this book will interest academics and can be used as a textbook in history or archives courses. It may also interest records managers and scholars in media studies and history, as well as data curation, art history, library science, and other disciplines.

